# Development of an mHealth App to Support the Prevention of Sexually Transmitted Infections Among Black Men Who Have Sex With Men Engaged in Pre-exposure Prophylaxis Care in New Orleans, Louisiana: Qualitative User-Centered Design Study

**DOI:** 10.2196/43019

**Published:** 2023-02-27

**Authors:** Meredith Edwards Clement, Aish Lovett, Sylvia Caldwell, Jeremy Beckford, Michelle Hilgart, Amy Corneli, Tabor Flickinger, Rebecca Dillingham, Karen Ingersoll

**Affiliations:** 1 Division of Infectious Diseases Louisiana State University Health Sciences Center- New Orleans New Orleans, LA United States; 2 Department of Psychiatry and Neurobehavioral Sciences University of Virginia Health Charlottesville, VA United States; 3 Department of Epidemiology University of Washington Seattle, WA United States; 4 Department of Population Health Sciences Duke University School of Medicine Durham, NC United States; 5 Duke Clinical Research Institute Durham, NC United States; 6 Division of General, Geriatric, Palliative, and Hospital Medicine University of Virginia Charlottesville, VA United States; 7 Division of Infectious Disease and International Health University of Virginia Charlottesville, VA United States

**Keywords:** pre-exposure prophylaxis, HIV, mobile apps, mobile phone

## Abstract

**Background:**

Sexual health disparities exist for Black men who have sex with men (BMSM) in New Orleans, Louisiana. Rates of sexually transmitted infections (STIs) are high for both BMSM and those taking HIV pre-exposure prophylaxis (PrEP).

**Objective:**

In this study, we introduced an existing PrEP adherence app to new potential users—BMSM engaged in PrEP care in New Orleans—to guide app adaptation with STI prevention features and tailoring for the local context.

**Methods:**

Using a user-centered design, we conducted 4 focus group discussions (FGDs), with interim app adaptations from December 2020 to March 2021. During the FGDs, a video of the app, app website, and mock-ups were shown to participants. We asked about facilitators of and barriers to STI prevention in general, current app use, impressions of the existing app, new app features to potentially facilitate STI prevention, and how the app should be tailored for BMSM. We used applied qualitative thematic analysis to identify themes and needs of the population.

**Results:**

Overall, 4 FGDs were conducted with 24 BMSM taking PrEP. We grouped themes into 4 categories: STI prevention, current app use and preferences, preexisting features and impressions of the *prep’d* app, and new features and modifications for BMSM. Participants noted concern about STIs and shared that anxiety about some STIs was higher than that for others; some participants shared that since the emergence of PrEP, little thought is given to STIs. However, participants desired STI prevention strategies and suggested prevention methods to implement through the app, including access to resources, educational content, and sex diaries to follow their sexual activity. When discussing app preferences, they emphasized the need for an app to offer relevant features and be easy to use and expressed that some notifications were important to keep users engaged but that they should be limited to avoid notification fatigue. Participants thought that the current app was useful and generally liked the existing features, including the ability to communicate with providers, staff, and each other through the community forum. They had suggestions for modifications for STI prevention, such as the ability to comment on sexual encounters, and for tailoring to the local context, such as depictions of iconic sights from the area. Mental health emerged as an important need to be addressed through the app during discussion of almost all features. Participants also stressed the importance of ensuring privacy and reducing stigma through the app.

**Conclusions:**

A PrEP adherence app was iteratively adapted with feedback from BMSM, resulting in a new app modified for the New Orleans context and with STI prevention features. Participants gave the app a new name, PCheck, to be more discreet. Next steps will assess PCheck use and STI prevention outcomes.

## Introduction

Rates of bacterial sexually transmitted infections (STIs) such as syphilis, gonorrhea, and chlamydia have increased substantially in the United States in recent years, and rates are particularly high in the Deep South, including in Louisiana [1]. These STIs disproportionately affect Black men who have sex with men (BMSM) [2]. Among BMSM, sexual risk behaviors including condomless anal intercourse are associated with social factors such as lack of peer support, medical mistrust, and self-reported experiences of homophobia or racism [3-7]. Our previous studies have aimed to better characterize social factors as potential barriers to STI risk reduction practices among BMSM using HIV pre-exposure prophylaxis (PrEP) [8,9]. Although we found moderate levels of perceived stigma and medical mistrust in this population, we also found that participants desired and were willing to engage in STI prevention strategies. Other previous studies have shown that interventions offering peer support can lead to reduction in condomless anal intercourse and number of male sexual partners among BMSM [10,11]. Mobile health (mHealth) apps offering peer support are a promising solution to facilitate the implementation of risk reduction strategies and potentially reduce STI incidence among BMSM who are prescribed PrEP. Furthermore, there is a high degree of interest in such apps with STI prevention features among men who have sex with men in the Southern United States [12].

An existing platform, *PositiveLinks*, is an mHealth app developed in 2013 to support viral suppression and retention in care among people with HIV [13]. *PositiveLinks* has several unique features including a community forum through which users can anonymously communicate to offer each other emotional support, pose and answer members’ questions, and share daily challenges or success stories [14]. It also allows for personalized, responsive interaction through the care team contact function, a feature of apps that has been noted to be desirable by PrEP clients [15].

*PositiveLinks* has previously been adapted to another app, *prep’d,* for the purpose of engaging clients in HIV prevention through PrEP. This study was undertaken to adapt the *prep’d* app to a new setting, taking into consideration the importance of understanding how the intervention’s design may function and appeal to a new population of users [15]. The objectives of this study were to introduce the *prep’d* app to new potential users in New Orleans, Louisiana; gather feedback about the app including peer support and proposed new STI prevention features; and iteratively modify the app based on feedback from participants.

## Methods

### Study Design

We conducted 4 focus group discussions (FGDs) with BMSM taking PrEP in New Orleans, Louisiana. We chose our sample size (N=4 FGDs) based on studies demonstrating when saturation is reached [16]. Using a user-centered approach, the app was iteratively modified between FGDs based on user feedback. Study activities were conducted from December 2020 to March 2021. FGDs were conducted remotely via Zoom (Zoom Video Communications, Inc) by a moderator who had undergone training in conducting qualitative studies. The FGDs lasted 1 to 2 hours and were audio-recorded.

### Participants

Participants were recruited via provider referral from our local PrEP clinics. Individuals were eligible to participate if they self-identified as a man who has sex with men, self-identified as Black or multiracial including Black, were aged 18 to 35 years at the time of recruitment, were engaged in PrEP care at 1 of 2 local PrEP clinics, and owned a smartphone. Demographic information was collected at the time of participation. Participants were assigned anonymous pseudonyms to protect their identity in the Zoom session and in the audio transcripts.

### Ethics Approval

This study was approved by the Louisiana State University Health Sciences Center–New Orleans institutional review board as an expedited protocol (#1457). An information sheet reviewing the study details was provided to all participants, and oral consent was obtained before participation. Participants’ names were not recorded on any data collection instrument. Each participant was assigned a unique participant identification number for use on all data collection materials.

### Data Management

Participant information (ie, name and mobile phone number) was kept in a separate, secure file and was only used for scheduling and compensation purposes. All hard copies of data generated during the study (eg, typed transcripts) were maintained in locked file cabinets or rooms, except when the data were being used. Electronic data files, such as typed transcripts and digital voice recordings, were stored on limited-access (ie, study team only) secure network drives at the Louisiana State University Health Sciences Center–New Orleans. All data shared with the University of Virginia (UVA) were uploaded to a secure file in UVA box, to which only the study personnel had access. Data received by UVA were stored on secure servers at UVA. To protect privacy, we also requested all participants to not repeat the information shared during the FGDs or any information related to the identity of other participants. Individuals were paid US $70 as compensation for their participation.

### FGD Content

During the FGDs, participants were first shown data from previous in-depth interviews (IDIs) with BMSM in which drivers of sexual risk taking and facilitators of and barriers to STI prevention were discussed [9]. Participants were asked to *debate and discuss* the findings from the IDIs including any topics that were controversial. Then, participants were asked about potential solutions to facilitate STI prevention among BMSM PrEP users. In addition, participants were asked about the current apps they used and which features of the apps were helpful. They were asked to comment on features to develop or modify for an app tailored for STI prevention among BMSM using PrEP. To gather feedback about the existing *prep’d* app, a 2.5-minute explainer video, mock-ups, and the *prep’d* app website were shown [17]. The initial *prep’d* app features include (1) daily self-monitoring check-ins for PrEP medication adherence (yes or no to pill taking), mood assessment (very happy, happy, OK, sad, and very sad), and stress level assessment (very low, low, medium, high, and very high); (2) Health Insurance Portability and Accountability Act–secure in-app private messaging with PrEP providers; (3) appointment reminders; (4) resources including PrEP clinic locator maps and *frequently asked questions* about PrEP; and (5) a community forum where participants could anonymously communicate with each other to provide social support. Participants were asked to comment on the current app and suggest modifications to assist with STI prevention and to better tailor the app for the New Orleans BMSM community.

### Analysis

After each FGD, our team met to conduct a rapid analysis to identify key findings and discuss feedback about the app, which were then incorporated into the app modification process. Prototypes and mock-ups of the newly proposed features were presented in the next FGD, and the process was repeated iteratively so that subsequent FGD participants reviewed the app content updated after previous FGDs. Subsequently, 3 think aloud sessions were conducted with participants, solely for usability purposes (findings are not discussed in this paper), and then, the final, new version of the app was submitted for production. Once all FGDs were completed, additional qualitative analysis of the FGDs using applied thematic analysis with coding using Dedoose (version 9.0.54; 2022) was performed [18]. A codebook was developed, informed by the interview guide and using an open coding approach and constant comparisons methodology. The codebook was refined iteratively, with 2 members of the study team independently coding each interview and resolving any discrepancies through consensus, until intercoder agreement was achieved. Final intercoder agreement was ≥70% for all FGDs. Themes were grouped into 4 categories: STI prevention, current app use and preferences, preexisting features and impressions of the *prep’d* app, and new features and modifications for BMSM. Descriptive statistics were used to summarize the demographic data.

## Results

### Demographic Characteristics

A total of 24 BMSM participated in the FGDs. Demographics are shown in Table 1. The median age was 28.2 (SD 4) years. Participants identified as gay or homosexual (19/24, 79%), bisexual (4/24, 17%), or queer (1/24, 4%). Most participants (18/24, 75%) were employed. Of the 23 participants, 10 (43%) had private insurance, 11 (48%) had Medicaid, and 2 (9%) were uninsured. Most participants (19/24, 79%) had been using PrEP for >12 months.

**Table 1:** Demographic characteristics of individuals who participated in the focus group discussions (N=24).

Characteristics			Values
Age (years), mean (SD)			28.2 (4)
**Race, n (%)**			
	Black or African American	21 (88)	
	Multiracial including Black or African American	3 (13)	
**Sexual identity, n (%)**			
	Gay or homosexual	19 (79)	
	Bisexual	4 (17)	
	Queer	1 (4)	
**Duration on PrEP^a^ (months), n (%)**			
	<3	2 (8)	
	3-6	2 (8)	
	6-12	1 (4)	
	>12	19 (79)	
**Employment, n (%)**			
	Employed and not in school	17 (71)	
	Employed and in school	1 (4)	
	Unemployed and not in school	5 (21)	
	Unemployed and in school	1 (4)	
**Education level, n (%)**			
	High school graduate or General Educational Development	5 (21)	
	Some college, technical, or vocational school	6 (25)	
	Technical or vocational school graduate	2 (8)	
	4-year college graduate	7 (29)	
	Some graduate school	2 (8)	
	Master’s degree or above	2 (8)	
**Housing status, n (%)**			
	Own or rent	16 (67)	
	Living with friend or relative	8 (33)	
**Income (US $), n (%)**			
	<15,000	7 (29)	
	15,000-49,999	12 (50)	
	50,000-74,999	5 (21)	
**Health insurance status (n=23), n (%)**			
	Private health plan	10 (43)	
	Medicaid	11 (48)	
	Uninsured	2 (9)	
**Male sex partners in the previous 6 months, mean (SD)**			3 (2.4)
	0-1, n (%)	5 (21)	
	2, n (%)	5 (21)	
	3, n (%)	8 (33)	
	4-5, n (%)	4 (17)	
	≥6, n (%)	2 (8)	

^a^PrEP: pre-exposure prophylaxis.

### STI Prevention

Subthemes for the STI prevention theme included concerns about STIs and suggestions for STI prevention.

#### Concerns About STIs

When reflecting on the IDI data, the FGD participants expressed that they were concerned about STIs and that anxiety and fear about some STIs were higher than that for others. A participant shared a personal experience and mentioned that PrEP had a positive impact on his sexual risk taking in that he had greater awareness of and knowledge about STIs now that he is on PrEP:

Years later when I moved to New Orleans and got on PrEP, I went from being tested once or twice a year to being tested every three months. So, to me, it kinda brought awareness of you’re being tested more frequently and also made me keep more account on how many sexual partners I’ve had within those three months. So, to me, it brought more education to myself and more awareness of, okay, say if you do get an STI, you can check who it is, instead of saying, “Well, I’ve only tested once this year so I don’t know who it could have been.”

Others described challenges to STI prevention among BMSM, such as general apathy about STIs as PrEP is being used to protect against HIV:

I feel like a little since PrEP came out, STIs aren’t talked about enough about how serious they can be even though they are treatable. And I feel like now people just kind of throw that out the window because now that they have PrEP, they have nothing to worry about.

Another participant shared the following:

But other than that, you walk in, you get treated, some places for free. You walk out, you wait just seven to 10 days and you’re back at it again. So, it’s just so convenient and fixable to get treated for an STI then take PrEP, which is why I’m taking PrEP. So, in general, most people think I’m taking PrEP, I don’t have to use a condom. And what’s the point of taking PrEP and using a condom when the condom is going to prevent it anyway? So, that’s kind of the mentality I see in a lot of people. So, it’s just so easy to get treated. It’s not that big of a deal. And since PrEP came out STIs are not that big of a deal anymore.

Other participants shared current norms around STI prevention; for example, they increased their condom use with new partners but decreased condom use with old or familiar partners. Another participant revealed his preconceived notions around STI risk—that he perceived risk based on the physical appearance or profession of his partners.

#### Suggestions for STI Prevention

Participants provided suggestions for ways to facilitate STI prevention among BMSM. Many desired the provision of resources, such as a tracker or dashboard to keep people informed about local or regional STI rates. They also wanted to know where they could get tested easily (eg, working hours and locations of testing sites). They desired education in a concise format and suggested symptom-based frequently asked questions or a place to get “straight to the point” questions answered. There was enthusiasm for an app to offer a sex diary, particularly for those who travel. A participant noted that “it would be really helpful” to have a calendar that showed PrEP pill taking and condom use relative to their sexual activity. A suggestion was to offer brief bits of information about STIs with visuals and to dissuade people from getting STIs by discussing the inconvenience of it and how one needed to wait a week after treatment to have sex again.

### Current App Use and Preferences

Subthemes for current app use and preferences included use of apps in general and use of health apps.

#### Use of General Apps

Participants from all FGDs (4/4, 100%) commonly used apps such as TikTok, Facebook, Instagram, Twitter, and Grindr and apps for their banks. They reported using apps to stay up to date with the times and to connect or reconnect with one another. A participant noted that all his apps were for “things that I use every single day, other than that I don’t need it on my phone.” Some were already using reminder apps such as those that provided a daily pill-taking reminder. Regarding the app features they liked, notifications were viewed to often be helpful but could also be “naggy.” A participant notably said that once he was annoyed enough to change the notification frequency, he would instead just delete the app from his phone:

Most [apps] do [allow users to modify the type and number of notifications], but if I have to go into my phone settings to modify the notifications, I just tend not to – not that I don’t want to, but usually I don’t think to do it until gets to the point where it’s an annoyance, but by then it’s already too late.

Participants desired apps that were easily maneuverable (eg, “fewer clicks”) and without too many advertisements:

I guess an app with simplicity, not too complex, just straight to the point stuff, where you don’t have to go around searching for stuff, it just kinda pops out at you.

Participants reported learning about the various apps they used through word of mouth, seeing the app on a friend’s phone, or advertisements on public transit and social media.

#### Use of Health Apps

Although some participants did not use health apps at all, other participants reported the use of health-related apps including meditation, mental health, and fitness apps. Some participants reported the use of pharmacy apps to order refills of their PrEP medication. Participants voiced support for health apps with reminders for appointments, refills, or taking daily pills. When discussing health apps specifically, they voiced support for a web-based chat feature through which they could easily communicate with their provider or care team.

Participants reported being unaware of any specific PrEP apps. When asked about the features that they would find useful for an app for PrEP users, participants requested frequent updates about new medications used or approved for PrEP and a resource guide for information about pharmacies, places to get tested, and ways to track sexual encounters. Participants also voiced interest in a way to verify PrEP use or offer proof that one really was taking or prescribed PrEP.

### Preexisting Features and Impressions of the prep’d App

Subthemes for features and impressions of the *prep’d* app included usability, esthetics and visuals, check-ins, community forum, and resources.

#### Usability of prep’d

After viewing the *prep’d* video or mock-ups, participants said that the app seemed to be usable and that they could see themselves using it. They commented that the app was well organized and not confusing. A participant noted that the app would be “beneficial towards our community” and would likely be used frequently. A participant stated the following:

I think I would – I think I would personally – I would use it for as long as I’m using the medication because I feel like even somebody said earlier, I just feel like both will go hand-to-hand.

Although others agreed that they would use the app persistently or as long as they were taking PrEP, some felt that the app would be more valuable to those who are new to PrEP, particularly in terms of the educational component (such as *PrEP 101*). A person noted that folks engaging with the app “in the beginning” would receive long-term benefits, but others were less sure about persistent impact:

Also, if someone’s very new to PrEP and don’t know really – they’re just interested in it. I want to say it’s a great learning tool for them to understand. But again, I wouldn’t know how long I would – because after a while, I wouldn’t see the use of it if I’ve been on PrEP for a year.

Participants had suggestions for how to increase longevity of use, for example, by continuously tweaking the app to keep users engaged and “constantly coming up with updates.” They also felt that clinical staff should promote the app and demonstrate its utility during PrEP visits with clients.

#### Esthetics and Visuals

A participant noted that the app was relaxing and looked similar to an app that would be used to book a vacation. Others felt that the color palette was boring and needed to be more “edgy.” A participant felt that the look should be more “nature-y” so that participants would be more inclined to share information or feelings. Some felt that the esthetics were critical to making the app appealing to them (eg, not “my scene” unless the visuals were just right), whereas others thought that the esthetics were not important and what really mattered was functionality:

Also, the colors are not sore to the eyes. Sometimes hard colors could be distracting. So, the color. The appeal. I’m not really concerned about aesthetics. I just like functionality. And it looks pretty functional to me from what I’ve seen.

A participant suggested making the background match the location, by including photos of the Superdome or the Mississippi Bridge to fit the New Orleans context. Others suggested allowing personalization of the app (eg, users should be allowed to choose their own color scheme and a quote of the day and have the ability to add or remove certain tracked health metrics from their display).

#### Check-ins

We demonstrated the daily check-in function in which users respond to questions about their medication adherence, mood, and stress. Some participants found these to be attractive, particularly noting that they could be good for one’s mental health:

And I love it for as far as mental health, recognizing your stress and different things like that. As someone who’s on that path of recognizing where I’m at in life, and taking my mental health more seriously, this will help me – this will help me in a convenient way to do that.

Some participants wanted the options to skip check-ins, whereas others thought they should not be skipped:

I don’t think it should be skipped myself, because I also – with the questions it gives a person a better ability to look at their habits. I think people can forget or just not really look into themselves and what they’re doing or what their behaviors are affecting them to do. You know? So, I do think having stress questions and the mood questions are beneficial. I don’t think they should be skipped.

They commented about the language of check-in feedback and initially felt that the language was robotic or similar to a “very old white man talking to me.” They wanted responses to their check-ins to be more enthusiastic, and they also desired variety. For example, if one reported high stress levels that day, a suggestion for the feedback was “stay strong” or “take a deep breath.” Another suggestion was for motivating language such as “keep it going, see you tomorrow.”

Participants liked the idea of check-ins for sexual activity and condom use, and thus, subsequent prototypes of the app included a question on daily sexual activity (sex with a condom, sex without a condom, or no sex). Some participants were enthusiastic about this additional feature of the app:

Yeah, I think it looks good, it’s simple. And it seems like it’s relatively easy to navigate. I like that you can add and take away certain metrics. You know, you can see, “Okay, what days did I have sex? The days that I did have sex, did I use a condom? What was my mood? Was I drinking that night?” Just all those things can kind of help you tell, you know, a story with that data, so I think a lot of helpful information.

Participants shared important feedback that the app’s initial responses to the sexual activity check-ins were judgmental and should be more sex positive, and changes to the app’s responses to the check-in answers were made accordingly.

#### Community Forum

Participants generally found value in the community forum feature. They felt that it could provide education, address stigma, build community, and allow users to share experiences. They liked the idea of having a support system through the forum:

For one, definitely the community app. It’s always good to share and get advice from people going through the same thing. That whole piece of being able to talk to someone anonymously about your issues and get an unbiased opinion about things that you may be going through can definitely help. Like I said, it can definitely – having that voice of reason that isn’t exactly someone you know can definitely help in certain situations.

A participant thought that it would be very valuable to “open up general lines of communication and help folks who are in situations that they feel unsure about.” The anonymous aspect of the forum was important; participants voiced that anonymous feedback and advice would be helpful, and they saw value in being able to say things that they would not normally tell others:

I think what’s important is just creating a safe space, so there is something that I would say among gay Black men that I probably wouldn’t say among a gay white man or a straight white woman or something like that who might be less knowledgeable and are still able to give their opinion.

The forum was also offered as a way to help users obtain quick answers to potentially embarrassing questions and alleviate any anxiety while they were awaiting a discussion with a health care provider about symptoms or side effects. A few participants voiced concerns about the forum, such as the potential for the spread of misinformation or the forum being used for bullying; frequent forum monitoring by study staff was offered as a way to prevent these from occurring. A participant worried that users may not often engage with the forum and recommended the need for a plan to keep people talking to each other.

#### Resources

Participants desired several resources through the app. They wanted alerts about STI outbreaks and repeatedly emphasized a desire to know current STI rates in their location; a participant labeled this function as “STI weather tracker app”:

I think factsheets would be really helpful, and this might, I guess, be a heavy lift for the developers, but if you could have some sort of data feed that would show, you know chlamydia rates in your area for the month of December. You know, just like what’s going on in your state and your city and your zip code.

They wanted direct or easy access to their health provider, such as a nurse hotline or phone number to reach someone who could answer health questions, automated chat, or live person to whom they could ask questions and have them answered. Regarding STI education, they preferred it to be in the form of a fact sheet or other easily digestible, concise format (“quick read” or “fun facts”). Participants felt that STI knowledge would be useful, particularly for those who “think that they’re invincible.” They voiced interest in a feature that would “tell you where to get tested,” and specifically, places that were accessible based on one’s location. They also supported the idea of having available pharmacies in the resource section:

I travel a lot, I work and I fly a lot just different places, like if I can’t get tested on my normal time just to be able to be like, “Hey, there’s a location here where you can get tested, these are the requirements,” because sometimes it’s hard to get to it.

In an FGD, participants discussed the need for mental health resources. They requested the contact information of mental health providers who were Black, queer, male, and affordable:

I was seeing a therapist and she was a Black woman, but I feel like she couldn’t get to me in the way that I feel a Black gay dude could get to me. So, I feel like having therapists that are in the community would definitely be a good thing to have on there.

Another participant mentioned the following:

And even better if you can find the ones that take Medicaid, because that too is a struggle. But I’ll take it – I’ll take whatever it can get it as long as it’s available because it’s already trying to find the last black unicorn.

### New Features and Modifications for BMSM

Subthemes for new features and modifications included sex diaries, discreet preference, and tailoring.

#### Sex Diaries

Participants liked the idea of sex diaries to allow them to quickly track their sexual encounters within the app. They felt that it was helpful to have a visual representation of recent activities and provided suggestions for the structure of the diaries:

Yeah. I definitely think this is an excellent function to have for more reasons than one. Some people’s sex lives are more active than others. And this app is about being safe and being aware and catching things – so, being preventive. So, having that tracking list is extremely beneficial for lots of reasons.

Another participant noted the following:

I personally tried to do it [track sexual activity] myself and I fail every time. But to have this app doing everything that I need in terms of my sexual activity, it’s awesome.

They wanted options to record the frequency of sexual encounters within the diary or calendar and to ensure that the diaries were confidential. They also desired a textbox to record their sexual partner’s contact information to get back in touch with them. They also felt that it would be useful to link their sexual activity to their daily mood and stress levels, and a participant noted the following:

Because I like feel it’s maybe good to quantify all this data for yourself to just track yourself, and your own behaviors. Because sometimes you can just be unaware that, oh, whenever I’m super-stressed, I also have sex that day. So, for me, I’d like to – I like that aspect of it.

In addition, they wanted to link their sexual activity to their PrEP use. They also thought that the diaries would be useful in answering providers’ questions about how many new or previous partners they had had since their last visit.

#### Discreet Preference

Participants voiced their preference for an app that was *incognito* and did not say “PrEP” anywhere. A participant provided an example of why PrEP should not be visible on the app:

Like when you’re with your grandmother or somebody, and she’s like, “What’s PrEP?” And you’re like, “Oh, nothing. Never mind.”

A participant noted that he would turn the notifications off if they showed up as “prep’d.” Participants were asked about their preferences for using a different name or label on the icon, and ultimately, the *prep’d* app was relabeled as *PCheck*.

#### Tailoring

Participants felt that if the app was tailored to BMSM, it should be more visually catered to their communities. Some advice to enhance BMSM ownership was to make the app feel as though “this was created for me” with great visual representation. A suggestion was to show scenes of Black men in normal social settings, such as going to dinner with friends or meeting with family members:

Just those different type of normal social settings that you would see a gay Black male in, making them feel comfortable in that, “Hey, this is where I belong. I can see myself in any one of these scenes.”

### The PCheck App

**Figure 1:**
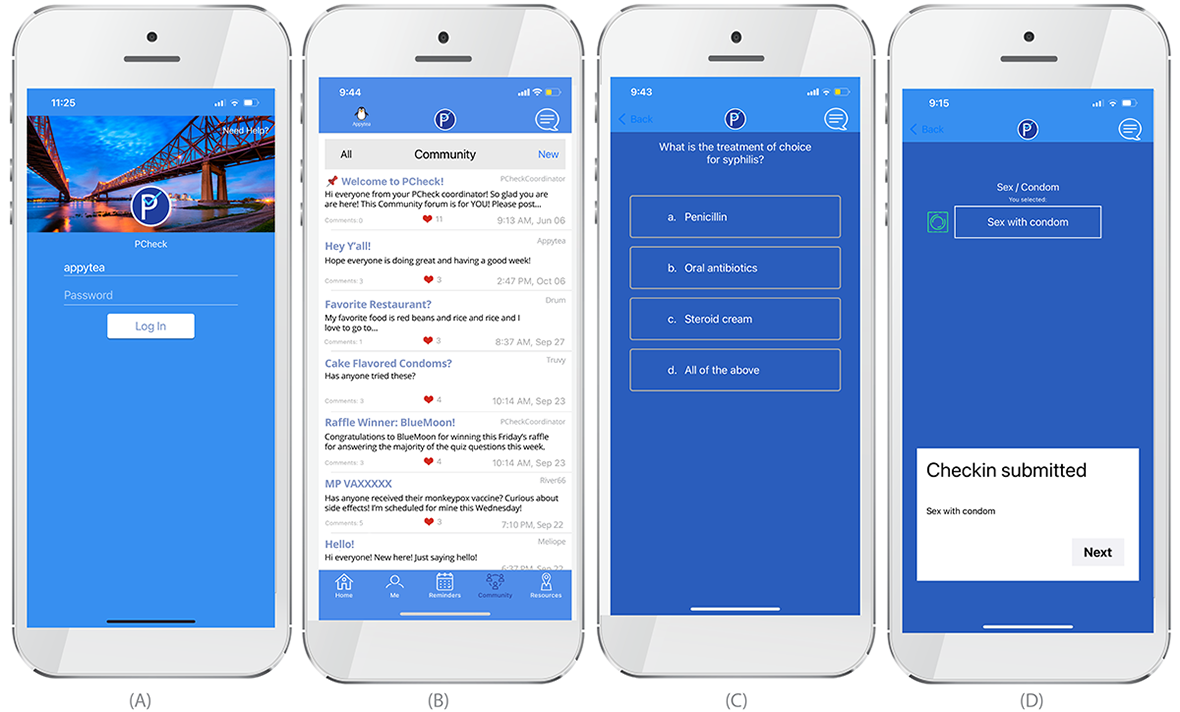
(A) New PCheck log-in featuring the Mississippi Bridge, (B) community forum, (C) daily quiz feature, and (D) daily check-in interface.

**Figure 2:**
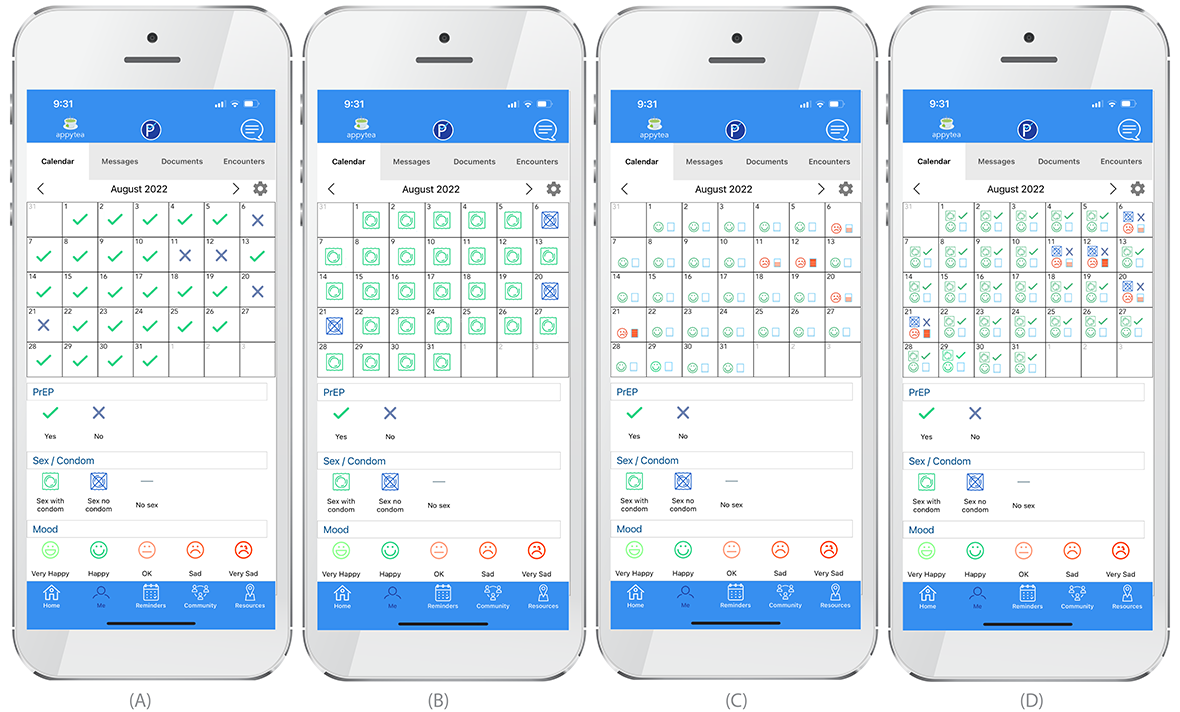
Daily check-ins, viewed individually (panels A, B, and C) and superimposed (panel D).

Guided by the findings from FGDs, a final version of the PCheck app (Figure 1) was developed, which includes some of the features of the original *prep’d* app along with modifications and additional features. The daily self-monitoring *check-in* for PrEP medication use, mood, and stress levels has been expanded to incorporate daily sexual activity and condom use, and each of these can be viewed individually or together on a monthly calendar (Figure 2). The app has retained Health Insurance Portability and Accountability Act–secure in-app private messaging with PrEP providers, which the participants of this study noted would be useful. The *PCheck* app will also retain the community forum, which was well liked by participants in our study (Figure 1). The *Resources* tab of the *prep’d* app has also been expanded; clinic locator maps will also include sites for STI testing and treatment. *Frequently asked questions* for STI symptoms and treatment, contact information of mental health providers, and updates on local events are also included. An additional educational feature is daily quizzes that ask about STI symptoms, transmission, and treatment (and sometimes, other trivia; Figure 1). Participants can also record specific sexual encounters, along with notes about the encounter (Figure 1).

## Discussion

### Principal Findings

We analyzed the focus group data obtained from BMSM engaged in PrEP care in New Orleans, Louisiana, and used the findings to modify and tailor an existing PrEP adherence app for the New Orleans context. Themes were transformed into app features through an iterative user-centered process. Through this formative study, we found that participants, in general, had a favorable impression of the adapted app, which they renamed *PCheck*. They believed that it would be straightforward to use and fitting with their preference for simple apps without too many notifications. Participants from all FGDs (4/4, 100%) reported that they would be inclined to use the app and found its features highly functional. They had suggestions for modifications to enhance esthetic appeal and usability.

Although some participants noted that those using PrEP are no longer intensely concerned with STI diagnoses, participants from all FGDs (4/4, 100%) desired and voiced their willingness to engage in STI prevention strategies through the app. Greater access to testing, STI education and fact sheets, sex diaries for tracking activity, and textboxes to write sexual partners’ information (to potentially facilitate contact tracing) emerged as potential strategies that have now been incorporated into *PCheck*.

Mental health emerged as an important need to be addressed through the app in the discussion about almost all features. Within the resources tab, participants wanted information about mental health providers; for the check-ins, participants desired the ability to track mood and stress levels; and regarding the community forum feature, participants endorsed the idea of a “safe space.” Encouragingly, the *PositiveLinks* app, from which *prep’d* and now *PCheck* have been adapted, has previously been shown to address mental health needs through direct messaging with local health care providers and by allowing for the delivery of valuable resources, particularly during the COVID-19 pandemic [19].

### Comparisons With Previous Studies

Previous studies have demonstrated that mHealth apps are appealing to BMSM and other men who have sex with men and are HIV-negative when they incorporate specified user-friendly features, including social networking and interactive engagement [20-22]. In addition, among BMSM, mHealth interventions have the potential to improve attitudes toward condom use and reduce episodes of condomless sex, particularly when peer support functions are included [10,11,23]. Peer support may also serve to enhance and sustain engagement [24]. Recent web-based studies have also shown the importance of psychosocial support among people who have or suspect they have an STI [25]. Our mHealth app, *PCheck*, is promising because it incorporates key features including the community forum for peer support; direct messaging with providers; and features that BMSM desired, including STI education and calendars for simultaneously tracking mood, stress, PrEP adherence, and sexual activity. In addition, the app has now been tailored to meet the needs of BMSM using PrEP within the New Orleans context, with local images and a new, more discreet name, *PCheck,* which was chosen by the FGD participants.

Compared with the development of completely new apps, adaptation of existing apps is a more cost-effective and time-saving approach that can maximize public health impact [26]. The initial *PositiveLinks* app was created for people living with HIV by a team of investigators at UVA and subsequently adapted for HIV prevention with the new name, *prep’d.* Our team in New Orleans further adapted the app by tailoring the content and incorporating new STI prevention features to *PCheck,* with the goal of facilitating PrEP adherence, retention, and STI prevention in our local setting. Such an adaptive process—to new populations and geographies and with engagement of local communities—allows for interventions that can more rapidly and practically respond to the *Ending the HIV Epidemic* initiative [26,27].

### Limitations

This study is limited in generalizability, given that we specifically recruited BMSM in the South as a demographic group subject to profound disparities in sexual health. In addition, smartphone ownership was required for participation, and therefore, our findings, including general appeal of the app intervention, may not be applicable to those who engage less frequently with smartphones or other web-based technologies.

### Conclusions

With the continued rise in rates of STIs across the country, prevention strategies are critically needed, including novel STI prevention strategies offered through mHealth. Given the almost universal smartphone ownership and frequent app use among young people in the United States, apps have tremendous potential to make an impact on public health [28,29]. We believe that the adaptation of our app according to end user feedback has resulted in a usable app that meets the needs of BMSM engaged in PrEP care. The *PCheck* app is currently being tested in a pilot randomized controlled trial (goal N=120 participants) to evaluate STI incidence and PrEP persistence in care (ClincialTrial.gov NCT05395754). If successful, this study would provide further evidence of the efficacy of mHealth apps to have an impact on sexual health and wellness. In addition, there would be potential for the app to be further adapted for other populations in other settings or contexts.

## Abbreviations

BMSM: Black men who have sex with men
FGD: focus group discussion
IDI: in-depth interview
mHealth: mobile health
PrEP: pre-exposure prophylaxis
STI: sexually transmitted infection
UVA: University of Virginia

## Data Availability

The data generated and analyzed during this study are available from the corresponding author upon reasonable request.
